# Linezolid-Induced Glossitis and Papillitis in Orthopedic Patients With Postoperative Infection: A Case Report

**DOI:** 10.1155/crdi/9036606

**Published:** 2025-08-10

**Authors:** Ryan J. Blake, Benjamin M. Frye, Allison M. Lastinger

**Affiliations:** ^1^Department of Orthopaedic Surgery, West Virginia University, Morgantown, West Virginia, USA; ^2^Musculoskeletal Infection Program, West Virginia University, Morgantown, West Virginia, USA; ^3^Department of Medicine, West Virginia University, Morgantown, West Virginia, USA

**Keywords:** arthroplasty, glossitis, infection, linezolid, orthopedics, papillitis

## Abstract

Linezolid, an oxazolidinone antibiotic, is commonly used to treat Gram-positive skin infections and has additional off-label success in treating bone and soft tissue infections. Linezolid has been associated with adverse effects, particularly those that are hematologic and neurologic in nature. However, we present three orthopedic patients who developed oral pain and swelling of the circumvallate papillae after a 10-day course of linezolid therapy, without the characteristic color changes associated with black hairy tongue as previously reported in the literature. All three patients were treated for superficial infections, and none exhibited signs of drug toxicity or neutropenia. Adverse side effects emerged despite short-term linezolid use, contrasting with previously reported cases involving long-term therapy. While black hairy tongue has been associated with linezolid and other antibiotics, these cases represent the first reported instance of generalized glossitis and papillitis without discoloration or noticeable alterations in the anterior portions of the tongue. The absence of coinfections or concurrent medications likely to cause similar symptoms suggests a unique set of side effects potentially correlated with linezolid. This case series emphasizes the importance of monitoring for atypical oral symptoms in patients on short-term linezolid therapy and adds to the growing body of literature on the side-effect profile. Further investigation into the mechanism of these reactions is necessary to better guide clinical practice in managing antibiotic-related oral adverse effects.

## 1. Introduction

Linezolid is classified as an oxazolidinone antimicrobial used in the treatment of Gram-positive infections with no coverage of Gram-negative organisms [1]. The oxazolidinone drug class is composed of synthetic drugs originally formulated to act as monoamine oxidase inhibitors for the treatment of depression [2]. However, later analysis of the drug class highlighted promising antimicrobial properties [2]. Multiple drug variations were developed with different potencies and side-effect profiles for coverage against Gram-positive organisms. Linezolid and tedizolid, another synthetic oxazolidinone, are the most favorable in terms of pharmacokinetics and adverse effects; they are the only drugs in the class with current US Food and Drug Administration approval. The clinical indications for linezolid specifically include bacterial pneumonia (nosocomial and community-acquired) and complicated/uncomplicated skin infections with off-label use for bone and joint infections [1]. The spectrum of the antibiotic includes vancomycin-resistant enterococci (VRE), coagulase-negative staphylococci, methicillin-sensitive *Staphylococcus aureus* (MSSA), methicillin-resistant *Staphylococcus aureus* (MRSA), *Bacillus* species, *Corynebacterium* species, and *Listeria monocytogenes* [3].

The mechanism of action for linezolid as an antibiotic involves the inhibition of protein translation through binding to the 23S ribosomal RNA. The drug binding interferes with the formation of the functional 70S initiation complex and prevents bacterial protein synthesis [4]. The drug exhibits bactericidal action against streptococcal species with only bacteriostatic activity against both staphylococci and enterococci. Interestingly, early periprosthetic joint infections (PJIs) tend to be caused by bacteria with high virulence such as *Staphylococcus aureus*, Enterococcus species, and coagulase-negative staphylococci, all of which respond well to treatment with linezolid including the off-label use for treatment of bone and joint infections [1, 5].

Orthopedic infections remain a clinical challenge due to their broad etiologies, and treatment can be complex involving both surgical and antimicrobial management. The risk of infection following orthopedic procedures is ever present due to the common use of foreign body implants. Superficial infection of a surgical site can potentially be managed with antibiotics alone, but deep infection involving implanted hardware requires revision surgery, an antibiotic regimen, and potential removal of infected hardware. However, in cases where prosthetic removal is contraindicated, clinicians may elect for long-term antibiotic suppression with retained hardware [6]. Linezolid remains a promising choice for the management of these surgical infections, and even with the positive side effect profile of linezolid, long-term use has been attributed to adverse effects. The most categorized adverse effects include blood dyscrasias (thrombocytopenia, anemia, and neutropenia), lactic acidosis, peripheral neuropathies, optic neuropathies, serotonin syndrome, and some rare reports of linezolid-associated black hairy tongue [1, 7] defined as a darkened discoloration of the tongue that may or may not be associated with a metallic taste.

We present three cases of orthopedic patients treated with linezolid for infection; these patients subsequently developed pain in the oral cavity with swelling of the circumvallate papillae. The patients admitted to discomfort and changes in taste like reports in the literature, but none exhibited black hairy tongue. To the best of our knowledge, these cases are the first reported adverse effects of generalized glossitis and swollen papillae with the lack of hairy black tongue following a course of linezolid antimicrobial therapy. All patients consented to be included.

## 2. Case Series

Case #1: A 71-year-old female with diagnosed osteoporosis underwent primary left total hip arthroplasty at our institution due to failure of conservative treatment and no improvement in hip pain. There were no complications with the procedure. She received perioperative vancomycin and cefazolin but was not discharged on any antimicrobial therapy. The patient endorsed a marked decrease in hip pain with improvement of symptoms following the total hip arthroplasty; however, approximately 1 month postoperatively, the incision site on the anterior thigh became edematous with slight wound dehiscence. The patient presented to our Joint Replacement Center Clinic for evaluation. The initial assessment of the incision was a superficial infection. Partial debridement of the wound was performed in the outpatient setting, but dehiscence appeared to track deeper than originally anticipated. Surgical debridement with wound revision in the operating room was performed. The patient was evaluated by the Musculoskeletal Infectious Diseases Service who prescribed a 10-day course of linezolid 600-mg oral tablet twice daily and ciprofloxacin 500-mg oral tablet twice daily to begin upon discharge for the debridement as cultures obtained in the operating room were pending. This regimen was chosen to provide empiric Gram-positive and Gram-negative coverages, respectively, while cultures were pending. After discharge, the operating room cultures grew *Morganella morganii* in four of five samples for which the strain was sensitive to ciprofloxacin. The original 10-day course of linezolid and ciprofloxacin was subsequently completed.

The patient returned for a 2-week follow-up with the Musculoskeletal Infectious Diseases Service. There was a lack of drainage from the incision site without any signs of infection. Antibiotics were finished 3 days prior to the follow-up, and the patient did not report any chills or fever. However, mouth discomfort was noted and, upon examination, there was inflammation of the tongue and enlarged circumvallate papillae in the posterior portions of the tongue consistent with generalized glossitis and papillitis. Laboratory tests were ordered to investigate potential drug toxicity (Table 1).

Case #2: A 68-year-old female presented due to a right thigh hematoma following right proximal femoral replacement surgery. The patient had a complex medical and surgical history resulting from a motor vehicle accident 14 years prior where she sustained a proximal femur fracture which was treated with a trochanteric fixation nail at an outside institution. Following a foot fracture on the ipsilateral side a few years later, the patient favored the right side and developed worsening pain with limited ambulation. After failing numerous rounds of conservative treatment, the patient established care at our institution for further evaluation of the hip. Plain radiographs of the right hip were obtained, and the previous hardware was found to be eroding into the acetabulum with an associated nonunion. After discussion with the patient about surgical options, the decision was made to move forward with surgical management with hardware removal, conversion to a total hip arthroplasty, complete proximal femoral replacement, and application of negative pressure wound therapy.

Due to the high risk for development of prosthetic joint infection, a 10-day course of oral doxycycline 100 mg was prescribed at discharge to a skilled nursing facility. The wound vacuum disconnected during the rehabilitation process while toileting, and the patient was unable to return to the clinic for reapplication of the unit due to contracting a viral illness. In the days following, a wound care nurse at the facility noted significant hematoma over the incision; the patient was brought to an outside facility and was subsequently transferred to our emergency department and ultimately admitted. A computed tomography scan of the hip demonstrated a hyperdense collection near the implant with concurrent acute kidney injury. Linezolid and piperacillin–tazobactam were immediately started to treat potential sepsis. The patient was taken to the operating room to drain the hematoma; during the procedure, cultures were obtained. Both the cultures from the operating room and blood cultures on admission showed no growth for 2 weeks. Infectious diseases recommended completing the course of linezolid and piperacillin–tazobactam with discharge back to the facility.

One-month follow-up with the Musculoskeletal Infectious Diseases Service did not reveal any signs of infection as cultures remained negative. The patient denied any fever or chills but mentioned changes in taste with associated soreness in the mouth. The tongue showed signs of inflammation with swelling toward the posterior aspect and no darkened discoloration. Repeat laboratory tests were performed at the clinic visit to investigate the potential for drug toxicity (Table 1).

Case #3: A 49-year-old male presented to our Joint Replacement Center Clinic with severe bilateral hip pain with the right reported worse than the left. The patient had exhausted all conservative treatments including physical therapy and joint injections, so the decision was made to undergo bilateral total hip arthroplasty procedures in a staged fashion beginning with the right side. The surgeries were planned approximately 3 months apart. The initial total hip arthroplasty and hospital course were unremarkable, and the patient was discharged home with no signs of infection.

The patient returned to the orthopedic clinic for a 2-week follow-up where he endorsed pain relief and improved function of the affected joint; however, there was dehiscence at the midportion of the incision. Inflammatory markers were raised at this visit, and there was no drainage from the incision. Oral cephalexin was started to treat a superficial infection, and the patient was instructed to return with any worsening of symptoms. Approximately 1 week later, the patient returned to the clinic where the wound continued to show signs of poor healing with superficial necrosis. Interestingly, inflammatory markers were within normal limits at the follow-up. The decision was made to admit the patient for a superficial wound debridement in the operating room with revision of the surgical incision. Intraoperative cultures were obtained during the procedure, and both daptomycin and cefepime were initiated until results were returned. Cultures were positive for *Serratia marcescens*, *Klebsiella pneumoniae*, *Staphylococcus epidermidis*, and *Corynebacterium aurimocosum*. The daptomycin and cefepime were discontinued, and linezolid and ciprofloxacin were started for a 10-day course per the recommendations of the Musculoskeletal Infectious Diseases Service.

**Figure 1:**
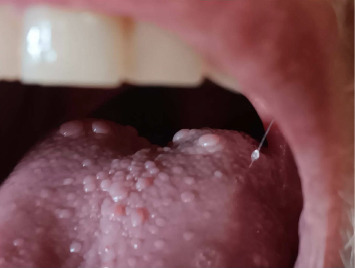
Swelling of the posterior portion of the tongue in Case #3.

The patient returned to the Musculoskeletal Infectious Diseases Service 2 weeks postoperatively for evaluation following the incision and debridement. Signs of infection were not present, and inflammatory markers were within normal limits (Table 1). The patient was able to tolerate all but the last two doses of antibiotics due to the development of tongue pain. It was noted on physical examination that the tongue was inflamed with marked swelling of the papillae, especially at the posterior border (Figure 1).

## 3. Discussion

Linezolid remains an effective treatment strategy when treating bone and soft tissue infections following orthopaedic procedures; the drug's efficacy has been demonstrated in many situations [8]. Linezolid's oral bioavailability and Gram-positive coverage make it an excellent choice when considering the high percentages of infections caused by staphylococci and streptococci species following bone and joint surgeries [8]. However, the complex nature of infection, especially when coupled with implantable hardware, creates a challenge for clinicians and patients. Depending on the clinical context, revision surgeries are commonly considered to remove potentially infected hardware; these cases often require concurrent antimicrobial therapy. Long-term suppression where revision surgery is not indicated or cannot be tolerated creates yet another layer of complexity for treating an infection. However, the cases presented here discuss potential adverse effects from linezolid that did not involve long-term suppression with the offending agent. In fact, all reported cases of generalized glossitis and papillitis developed within a 10-day course of antibiotics.

A correlative relationship has been made between the length of treatment with linezolid and the incidence of adverse effects [9]. Preclinical toxicology exhibited mild yet reversible alterations in both hepatic and hematopoietic function, and some authors have reported patients developing pancytopenia within a month and complete recovery within 3 weeks after abrupt cessation [9, 10]. The most frequent adverse effects after the preclinical phase included diarrhea, nausea, vomiting, and tongue discoloration (0.2% of the cases) [11–13]. Like the reports in the literature of myelosuppression, discontinuation of the agent resulted in complete resolution of the symptoms, including tongue discoloration. Additional cases reported in the literature have further defined the tongue discoloration as a rare condition known as black hairy tongue.

The mechanism of black hairy tongue following linezolid use is somewhat unclear. The finding is characterized by black-to-brown discoloration with less frequent reports of white, yellow, and even green [14]. Most patients deny pain associated with the condition and seek evaluation for cosmetic purposes. The underlying mechanism has been hypothesized to involve changes in the intraoral environment catalyzed by the antimicrobial therapy that promotes the growth of specific microorganisms. The presence of these organisms can potentially alter the desquamation process of the dorsal surface, furthering the symptomatic discoloration while creating the hair-like appearance of the filiform papillae [14]. Dermoscopic examination of black hairy tongue has also been shown to have characteristic features of the filiform papillae resembling that of a sea anemone, which differentiates it from other manifestations in the oral cavity [15].

All the cases at our institution were most closely related to linezolid-induced black hairy tongue but only in relation to the affected tissue. The patients did not show any signs of abnormal discoloration that would align with already reported adverse outcomes. Eruptive lingual papillitis has been previously described as an inflammatory process affecting the fungiform papillae with an associated hypertrophy [16]. However, eruptive lingual papillitis tends to describe inflammation confined to the anterior and lateral portions of the tongue [16]. Generalized glossitis described herein was noted throughout the entire tongue while papillitis, defined as swelling of the papillae, was confined to the posterior aspects near the circumvallate, and not the fungiform, papillae. In addition, black hairy tongue is characterized by alterations of the anterior portions of the tongue involving the filiform papillae. These findings represent major differences between the patients presented here and those in the literature.

Black hairy tongue is not unique to linezolid use, however. Other antibiotics have been shown to cause these findings: amoxicillin clavulanate, metronidazole, doxycycline, erythromycin, tetracycline, moxifloxacin, imipenem/cilastatin, and piperacillin–tazobactam [14, 17]. Interestingly, one patient who developed generalized glossitis and papillitis was prescribed both doxycycline and piperacillin–tazobactam, but again, the presentation was not descriptive for black hairy tongue. The other two patients were prescribed ciprofloxacin with linezolid. Ciprofloxacin use has shown only mild adverse effects at therapeutic doses mostly limited to the gastrointestinal tract [18], making the adverse outcomes less likely to be caused by ciprofloxacin specifically. The combination of the two drugs has not been shown to cause mouth soreness or inflammation consistent with these patients either. Aside from medication use, infection of the oral cavity could be a causative mediator explaining the symptoms. However, neutropenia and immunodeficiencies were absent, making recurrent infection less likely. In short, the three patients discussed herein appear to have had unique adverse effects from linezolid used to treat their superficial infections with complete resolution of oral symptoms within less than a month following cessation of the antimicrobial agent, which highlights a promising prognosis.

There does appear to be a temporal association between this specific antibiotic therapy and the development of adverse side effects in these patients. However, it is important to note potential limitations of the correlative, and not necessarily causative, relationship of oral symptomology and administration of linezolid. Drug-induced effects are diagnoses of exclusion, and there remains a possibility that concurrent medication use was underlying the pathogenesis. In the three cases presented here, there were no combinations of drug regimens, other than antimicrobial agents discussed previously, that were introduced around the time of symptom presentation that would explain the findings based on current literature. In addition, it does remain possible that other disease processes may be responsible for the mucosal manifestations, but no other systemic, concurrent infectious conditions or nutritional deficiencies were documented. This report should serve as a general framework aiding in the surveillance of adverse effects associated with linezolid.

Linezolid is an antimicrobial agent for Gram-positive organisms that has shown success in many contexts, including in orthopedics for bone and soft tissue. The drug is generally well tolerated with the most common adverse effects involving gastrointestinal upset, blood dyscrasias, and neuropathies limited to those on long-term therapy with an associated reversal following drug cessation. Some rare reports of black hairy tongue have been described with this medication, but to our knowledge, there are no reports of oral pain and swelling while using linezolid in an acute setting. The side effects presented here reflect a potential novel adverse effect compared with other oral manifestations reported in the literature. Potential mechanisms explaining these findings could include direct toxicity to mucosal surfaces [19], disruption of a microbiome with subsequent inability to regulate pathogenic insult [20], or allergic responses in predisposed individuals [21]. However, further monitoring is necessary to quantify these reactions while strengthening its association in the setting of acute linezolid use.

## Figures and Tables

**Table 1:** Laboratory measures when adverse outcomes were noted with abnormal results bolded.

Laboratory test	Laboratory measure	Case #1	Case #2	Case #3
Inflammatory markers	C-reactive protein (mg/L)	5.0	**20.0**	3.2

Complete blood count	White blood cell (10^3^/μL)	9.2	7.1	8.1
	Hemoglobin (g/dL)	12.6	**7.9**	**12.5**
	Hematocrit (%)	39.5	**26.3**	**37.6**
	Platelet count (10^3^/μL)	229	253	253
	Red blood cells (10^6^/μL)	4.54	**2.69**	**3.99**
	Mean corpuscular volume (fL)	87.0	97.8	94.2
	Mean corpuscular hemoglobin concentration (g/dL)	31.9	**30.0**	33.2
	Mean corpuscular hemoglobin (pg)	27.8	29.4	31.3
	Red cell distribution width coefficient of variation (%)	14.8	**19.2**	14.4

Differential	Polymorphonuclear cells (%)	65.2	58.9	61.0
	Lymphocytes (%)	18.3	23.1	24.0
	Eosinophil (%)	2.8	9.8	4.0
	Monocytes (%)	12.3	7.0	10.0
	Basophils (%)	0.5	0.6	1.0
	Immature granulocyte (%)	0.9	0.6	0.0
	Immature granulocyte (10^3^/μL)	< 0.10	0.04	< 0.10
	Polymorphonuclear cells (10^3^/μL)	5.96	4.21	5.04
	Lymphocytes (10^3^/μL)	1.68	1.65	1.90
	Eosinophils (10^3^/μL)	0.26	**0.7**	0.28
	Monocytes (10^3^/μL)	**1.13**	0.50	0.82
	Basophils (10^3^/μL)	< 0.1	0.04	< 0.10

Chemistry	Blood urea nitrogen (mg/dL)	20	18	17
	Creatinine (mg/dL)	0.82	0.87	0.85
	Glomerular filtration rate (mL/min/BSA)	76	> 60	> 90
	Albumin (g/dL)	4.0	**3.1**	3.9

Liver/pancreas function	Bilirubin, total (mg/dL)	**0.2**	0.4	0.3
	Bilirubin, conjugated (mg/dL)	0.1	0.2	0.1
	Aspartate transferase (U/L)	**48**	31	22
	Alanine transaminase (U/L)	22	50	26
	Alkaline phosphatase (U/L)	93	85	73

*Note:* Bold values are outside the normal reference range.

## Data Availability

The datasets used and/or analyzed during the current study are available from the corresponding author upon reasonable request.
